# Selective Vulnerability to Tau Pathology in Serotonergic Dorsal Raphe Nucleus Neurons

**DOI:** 10.1002/alz.091738

**Published:** 2025-01-03

**Authors:** Benjamin J Cooper, Mikayla L Hunter, Samantha R Pierson, Catherine A Marcinkiewcz, Marco M Hefti, Kimberly L Fiock

**Affiliations:** ^1^ The University of Iowa, Iowa City, IA USA; ^2^ University of Iowa, Iowa City, IA USA

## Abstract

**Background:**

The dorsal raphe nucleus (DRN) is the primary source of serotonergic projections to supratentorial structures. We and others have shown that it is selectively vulnerable to tau pathology in both human and mouse models of early AD. Although well characterized in mice, the neurochemical anatomy of the human DRN, and in particular the role of Vesicular glutamate transporter‐3 (VGLUT3)‐expressing neocortical projection neurons in tau pathology, remains unclear.

**Method:**

Post‐mortem human brain tissue was obtained from previously consented cases stored in the Iowa Neuropathology Resource Laboratory. Next of kin provided consent for research use of tissue. Sections were processed, embedded in paraffin, and sectioned in the usual fashion. Tyrosine hydroxylase (TH), tryptophan hydroxylase 2 (TPH2), and phosphorylated tau (AT8) were visualized by immunofluorescence, while VGLUT3 transcripts were identified by RNA in situ bybridization. Imaging was done using a Cytation5 platform with downstream analysis using MATLAB and R.

**Result:**

We found that, similar to mice, the human dorsal raphe has both VGLUT3‐positive and –negative serotonergic neurons. Unlike mice however, in the human DRN, these are not anatomically separate, and the proportion of TPH2 neurons that are VGLUT3 appears to be higher than in mice. In patients with DRN tau aggregates, these appear to selectively affect TPH2+/VGLUT3+ neurons.

**Conclusion:**

Serotonergic neurons in the dorsal raphe express high levels of VGLUT3, with an anatomic distribution differing from that seen in mice, and these neurons appear to be selectively vulnerable to tau pathology.

